# Motion corrected MRI differentiates male and female human brain growth trajectories from mid-gestation

**DOI:** 10.1038/s41467-020-16763-y

**Published:** 2020-06-16

**Authors:** Colin Studholme, Christopher D. Kroenke, Manjiri Dighe

**Affiliations:** 1 Biomedical Image Computing Group, Department of Pediatrics, University of Washington, Box 356320, 1959 NE Pacific Street, Seattle, 98195 WA USA; 2 Department of Bioengineering, University of Washington, 1959 NE Pacific Street, Seattle, 98195 WA USA; 3 Department of Radiology, University of Washington, 1959 NE Pacific Street, Seattle, 98195 WA USA; 4 Advanced Imaging Research Center and Division of Neuroscience, Oregon National Primate Research Center, Oregon Health Sciences University, 3181 SW Sam Jackson Park Road, Portland, 97239 OR USA

**Keywords:** Magnetic resonance imaging, Image processing, Machine learning, Neuroscience, Development of the nervous system

## Abstract

It is of considerable scientific, medical, and societal interest to understand the developmental origins of differences between male and female brains. Here we report the use of advances in MR imaging and analysis to accurately measure global, lobe and millimetre scale growth trajectory patterns over 18 gestational weeks in normal pregnancies with repeated measures. Statistical modelling of absolute growth trajectories revealed underlying differences in many measures, potentially reflecting overall body size differences. However, models of relative growth accounting for global measures revealed a complex temporal form, with strikingly similar cortical development in males and females at lobe scales. In contrast, local cortical growth patterns and larger scale white matter volume and surface measures differed significantly between male and female. Many proportional differences were maintained during neurogenesis and over 18 weeks of growth. These indicate sex related sculpting of neuroanatomy begins early in development, before cortical folding, potentially influencing postnatal development.

## Abstract (web summary)

The human fetal brain may exhibit early sex differences. Here, the authors present MRI based analysis female and male brain growth. Cortical development follows very similar trajectories at larger scales, while differing in focal regions. White matter growth differs at both large and small scales.

## Introduction

Accounting for sex differences in biomedical research is increasingly important both on a clinical and basic science level^1^. Several neurodevelopmental diseases, such as Autism and schizophrenia, exhibit sex differences in incidence, age of onset, and symptomatology^2,3^. Additionally, male and female fetal brains are differentially vulnerable to adverse intrauterine exposures^4^, as well as to premature birth, and associated perinatal brain injury^5,6^. A more complete understanding of the development of sex differences is needed to address this important factor in neurodevelopmental diseases.

Differences between adult male and female human brains have been observed in numerous magnetic resonance imaging (MRI) studies and confirmed on increasingly larger populations^7^. Many sex differences are thought to arise during the critical period of postnatal development when hormones act on brain structural organization^8^, and may also further influence brain anatomy through puberty and adult life^9^. Human studies have also examined the separate effects of hormones and sex chromosomes on brain development^10^.

Prior to the postnatal impact of hormones, earlier differences are believed to originate, at least in part, from differential exposure to androgens during fetal growth^11^, and also differences in gene expression^12^. Fetal levels of testosterone are highest in males by 18 weeks gestation^13,14^ and may remain high through to 24 weeks^15^. Recent twin studies have also highlighted the possible influence of fetal testosterone on later development^16^. However, because of the challenge of accurately quantifying human fetal brain development before birth using techniques such as ultrasound and MRI, the limited number of early small cross-sectional studies at lower resolution has not reported clear prenatal neuroanatomical differences in global or regional brain tissue growth.

It is important to characterize potential sex differences in brain structure in the prenatal period because the dynamic neuroanatomical development observed throughout the postnatal lifespan^17^ indicates that differences change, and therefore may also be potentiboally very different before birth than those reported postnatally. Further, several developmental processes, including cell proliferation, migration, and differentiation, occur only within the fetal period, and assessment of structural differences during this developmental period could provide important and fundamental mechanistic information related to structural and functional differences observed postnatally and at maturity.

Studies of later postnatal neuroanatomy using MRI^18–21^ indicate that some neuroanatomical sex differences are detectable 1 month after birth, but have not explored their change over time, and it is not clear if they emerge during the 4 weeks of growth after birth. Morphometry of clinically prenatal brain anatomy has been the first route to studying brain development before normal term age, allowing, for example, the study of late gestation cortical folding^22^. However, these studies have not reported evidence for statistically significant sex differences in absolute volume or brain surface features before term age^23^. Such studies are limited both in size and more specifically, due to their clinical cohorts, in their ability to reflect true normal prenatal growth. Small-scale in utero MRI studies have been limited to whole brain tissue measures, using early motion correction techniques, and have either not found^24,25^ or not reported^26^ statistically significant sex differences before birth. They have also not been able to use repeated measurements in each subject.

**Fig. 1:**
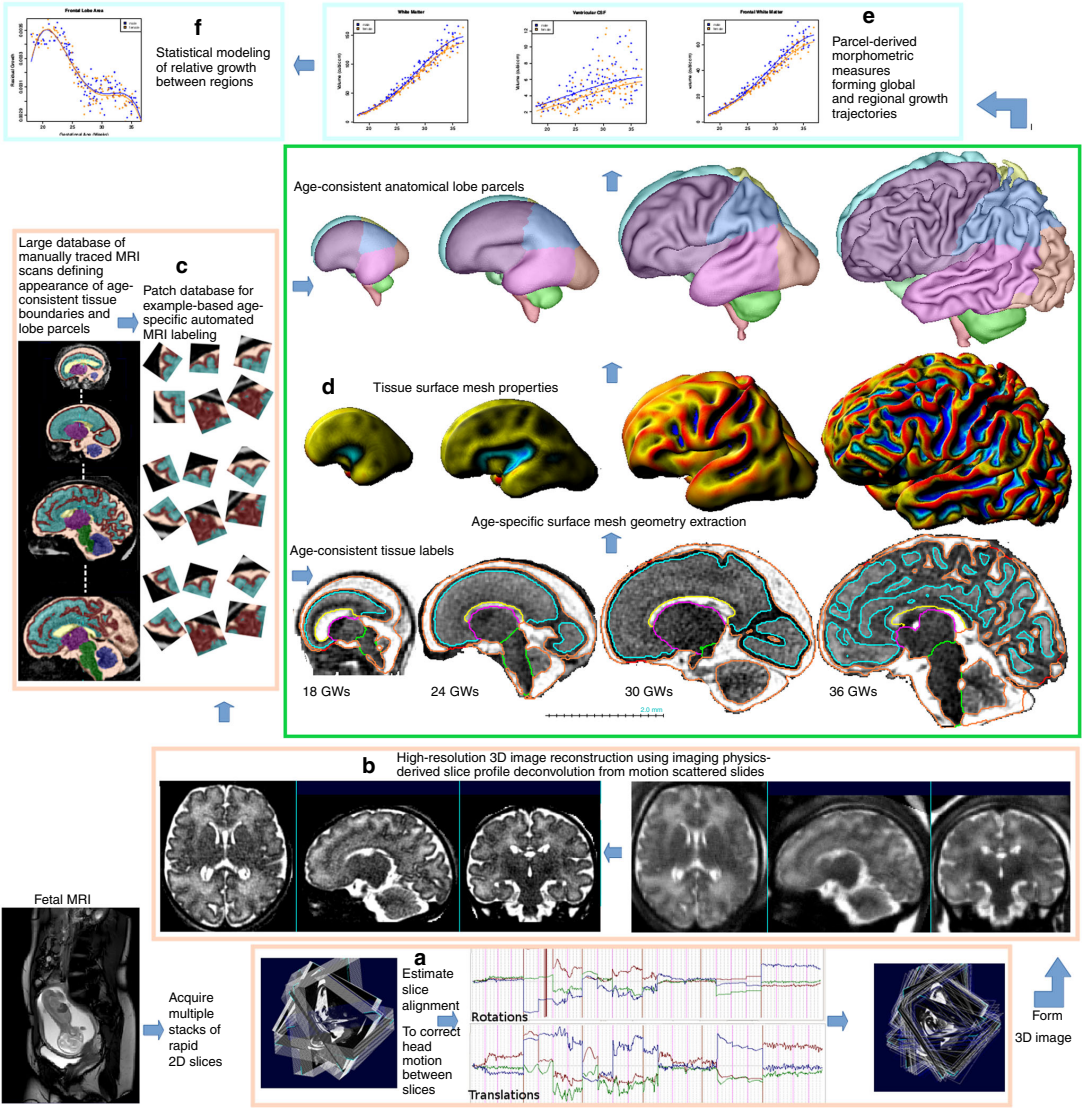
Combining computationally assisted imaging and machine learning-based analysis allows large-scale studies of normal healthy volunteer pregnancies over a long gestational range with a high yield. Accurate motion correction (**a**) of fast multi-planar multi-slice MRI data^27,56^ allows high spatial resolution (0.5 mm cubic voxels) 3D image (**b**) reconstruction^29^. Databases of expert delineated 3D images allow accurate and fully automated example-based tissue segmentation (**c**) into age-consistent classes, together with parcellation into lobe regions that can be tracked over long periods of development. Creation of surface representations at high resolution (**d**) allows mapping of subtle surface properties, such as local area and curvature to accurately quantify cortical development. Mixed-effects modeling of repeated measures (**e**) from fetuses allows fitting of non-linear growth models and analysis of complex relative growth trajectories between local and global regions (**f**).

Continuing advances in fetal MRI and computational image reconstruction^27,28^ enable increasingly robust and higher-resolution 3D fetal brain imaging to be reliably acquired repeatedly throughout the second half of gestation. In particular, these computational imaging techniques employ robust super-resolution deconvolution to combine motion scattered slice data, allowing high isotropic resolution image reconstruction^29^ from thick imaging slices acquired in different orientations (Fig. 1). Critically, these techniques dramatically improve the rendition and quantification of cortical tissues in the early fetal brain. These imaging advances are combined with a machine learning-based image analysis framework, leveraging the latest high-resolution expertly delineated neuroanatomical training database, specifically designed for the developing human fetus. These techniques allow fully automated and precise extraction of developmentally consistent tissue boundaries and anatomical regions over 18 gestational weeks (GWs). These consistent regional anatomical parcellations allow not just quantification of global measures of brain anatomy, but also lobe scale and millimeter scale mapping of growth patterns over 18 GWs as the brain is sculpted during neurogenesis, cell migration, and cortical folding.

This study provides an analysis of the first large-scale volumetric dataset with repeated measurements derived from high-resolution 3D images, to significantly extend the current postnatal characterization of structural differences between male and female brains to before the mid-gestation fetal period. These data reveal the presence of a number of structural differences and similarities in the male and female brain as early as 18 GWs that were previously reported in adults and children, and also characterizes their change over long periods of early brain growth.

## Results

### Data

The study cohort recruitment and inclusion/exclusion criteria are described in detail in the “Methods” section and we include overall subject and scan demographics in Table 4, which includes statistical tests for possible differences in the male and female groups that were not related to imaging measurements. Fetal-specific MRI, image reconstruction, and image analysis methods using automated tissue labeling and parcellation as illustrated in Fig. 1 are described in detail in the “Methods” section. The brain tissues in the 3D MRI data were divided into cortical (developed gray matter or earlier cortical plate) and non-cortical (developed white matter or earlier transient developmental zones^25^), deep gray matter, and cerebellum regions. These were specifically defined to have boundaries that were consistently identifiable in MRI over the entire developmental period to allow tracking of growth. The brain was also automatically parcellated into lobe regions consistent over the entire age range studied to create measures of global and regional tissue volume, area, and surface curvature. All these data were then analyzed using mixed-effects models to incorporate information about within- and between-subject variance when modeling the growth trajectories. Because of the challenge of imaging busy working pregnant volunteers at particular intervals and with a consistent number of repeated scans, the data were analyzed in an adaptive unbalanced statistical mixed-effects framework to allow the use of data from subjects being able to contribute more or less scans during their pregnancy.

### Absolute growth trajectories

**Fig. 2:**
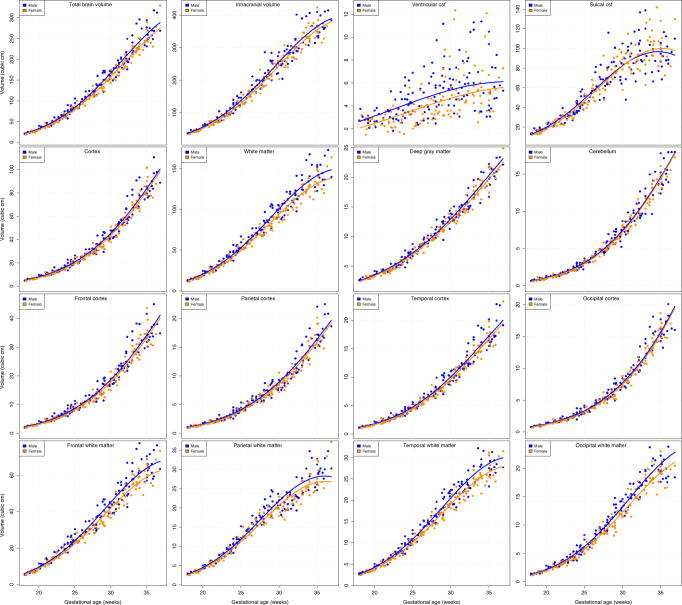
Plots of global and lobar absolute volume measures against age. Measures for male and female fetuses together with the fitted models in each class transformed to measurement units for visualization. Non-linear growth trajectories over the 18 weeks are apparent for all the measures. Differences in male and female measures are apparent and increase with age, but analysis shows that this difference follows a constant fractional ratio between male and female (see Table 1). The smallest sex differences in tissues are seen in the cerebellum volumes, which do not reach statistical significance.

Tissue volume measures and resulting model estimates are summarized by the graphs in Fig. 2, which reveal accelerating, non-linear growth in the measures over the 18-week period. Rates of increase of white matter, ventricular cerebrospinal fluid (CSF) (VENT), and sulcal CSF (SCSF) volume begin to fall off on approaching 36GW, while cortex, deep gray matter, and cerebellum continue to maintain or increase in their rate of growth toward normal term age. All variances also visibly increase with age (as discussed in the “Methods” section), as individual variability between subjects accumulates during development. However, SCSF and VENT exhibit much higher relative variance than the tissue volumes. The male and female trajectories fitted to the cortex are clearly much closer in relation to their variance, both globally and for individual lobes, than they are for white matter volumes.

Statistically, as summarized in Table 1, different absolute growth trajectories were detected in male and female fetuses for almost all the measures, with those globally and regionally related to white matter exhibiting the most dramatic and statistically significant differences. Expressed as percentage differences, white matter was 7.38% larger in males, whereas cortical volume was only 5.08% larger. Cortical volume differences were greater in males globally, but, unlike white matter, many of these differences did not survive correction for multiple comparisons globally or in many of the lobe regions. However, the temporal cortex exhibited a 5.09% greater volume in males over the gestational age range that did survive correction for multiple comparisons. Deep gray matter regions were larger in males by 3.52%, but this did not survive correction for multiple comparisons. Cerebellar volumes were significantly (uncorrected only) larger in males by 3.40%. Surrounding the brain, global SCSF and VENT volume exhibited much greater variance in comparison to tissue volume measures (seen particularly in the plots). As a result, although ventricular volume was overall statistically greater in males compared to females, by 17.96%, there was a very wide 95% confidence interval (>23%) in the actual estimate of this percentage difference.

**Table 1:** Differences in absolute growth trajectories.

R: Region	*R*_M_ − *R*_F_	Confidence interval		*P* value	*P* value
Measure	@ 27GW	−2.5%	+2.5%	(M∣F)	(M∣F) × AGE
TBV	6.24%	3.48%	9.55%	**9.54e** − **06**^†^	4.38e − 01
AREA	3.68%	1.34%	5.87%	**2.84e** − **03**	3.25e − 01
CURV	−2.20%	−3.98%	−0.35%	**1.87e** − **02**	4.33e − 01
ICV	5.53%	2.89%	8.36%	**1.00e** − **04**^†^	**1.84e** − **02**
CORT	5.08%	1.86%	8.22%	**1.50e** − **03**^†^	5.88e − 02
WM	7.38%	4.55%	10.22%	**6.20e** − **07**^†^	9.49e − 01
DGM	3.52%	1.20%	5.81%	**4.79e** − **03**	6.90e − 01
CEREB	3.40%	0.04%	6.94%	**4.06e** − **02**	9.88e − 02
VENT	17.96%	6.29%	29.82%	**9.32e** − **04**^†^	1.23e − 01
SCSF	2.67%	−1.57%	7.58%	2.69e − 01	**7.24e** − **03**
Frontal CORT	5.38%	1.69%	8.53%	**2.42e** − **03**	1.11e − 01
Parietal CORT	4.95%	1.77%	8.53%	**3.25e** − **03**	2.40e − 01
Temporal CORT	5.09%	2.14%	8.20%	**9.70e** − **04**^†^	8.23e − 02
Occipital CORT	4.40%	0.92%	8.46%	**1.05e** − **02**	5.59e − 02
Frontal WM	7.63%	4.20%	11.10%	**4.33e** − **06**^†^	7.54e − 01
Parietal WM	5.80%	2.74%	8.62%	**1.61e** − **04**^†^	7.17e − 01
Temporal WM	8.01%	5.33%	10.85%	**4.41e** − **08**^†^	7.44e − 01
Occipital WM	8.02%	4.55%	11.30%	**5.05e** − **06**^†^	8.42e − 01
Frontal AREA	4.06%	1.17%	6.60%	**2.59e** − **03**	7.26e − 01
Parietal AREA	2.70%	−0.05%	5.55%	**3.45e** − **02**	8.87e − 02
Temporal AREA	4.54%	1.84%	7.11%	**2.72e** − **04**^†^	4.50e − 01
Occipital AREA	3.46%	0.30%	6.35%	**2.41e** − **02**	2.31e − 01
Frontal CURV	−1.75%	−3.72%	0.34%	8.59e − 02	3.40e − 01
Temporal CURV	−1.37%	−2.84%	−0.10%	**4.02e** − **02**	5.29e − 01
Parietal CURV	−2.76%	−5.49%	0.64%	7.46e − 02	4.64e − 01
Occipital CURV	−3.71%	−5.67%	−1.58%	**2.73e** − **04**^†^	5.19e − 01

The estimated percentage increase from the female to male fetal brain of the absolute growth of regional anatomical measures estimated (see also Fig. 2), and the upper and lower 95% confidence bounds on this difference estimate.

*TBV* total brain tissue volume, *ICV* intracranial volume, *CORT* cortical tissue volume, *WM* combined white matter/developing white matter tissue volume, *DGM* combined deep gray matter volume, *CEREB* cerebellum volume, *VENT* ventricular cerebrospinal fluid volume, *SCSF* sulcal cerebrospinal fluid volume, *CURV* mean surface curvature averaged over the specified portion of the brain surface, *AREA* surface area of total brain or lobe region.

In addition, we show the statistical significance (uncorrected *P* value) of the difference in the growth models and the significance of the change of this with age. Bold indicates *P* < 0.05 for the male − female trajectory difference and dagger indicates this significance survives correction for multiple comparisons across the 26 two-sided hypothesis tests in this table.

No statistically significant sex–age interaction was seen either before or after correction for multiple comparisons in any of the basic tissue volume estimates, indicating that the fractional difference from female to male fetuses remained constant as all measures for the different regions increased. There was however an age interaction in intracranial volume (ICV), which appears therefore to be driven by CSF effects. Although SCSF was not overall significantly different between males and females, there was a significant age interaction with SCSF volume that did not survive Bonferroni correction, together with an (uncorrected) significant age interaction in overall ICV. The variance in the baseline CSF measures (with confidence intervals ranging from −1.57% to +7.58% in SCSF) makes it more difficult to interpret these findings.

The absolute brain surface area measurements were statistically greater in males over the whole brain (not surviving correction for multiple comparisons) and regionally in each lobe. They were most consistently different in the temporal lobe where the effect remained significant after correction for multiple comparisons. Mean surface curvature (calculated as described in the “Methods” section) was statistically (uncorrected) greater in females averaged over the whole brain and over the temporal and occipital lobes. The occipital lobe showed a more consistent difference in curvature with greater average absolute mean curvature in females compared to males after correction for multiple comparisons. This greater curvature in the female fetal brain seemingly reflects the smaller tissue volume and surface area (which induce a higher average absolute mean curvature on the surface), implying that surface shape and relative folding may be consistent, and curvature is simply changing with overall brain size differences.

### Relative (residual) growth trajectories

**Fig. 3:**
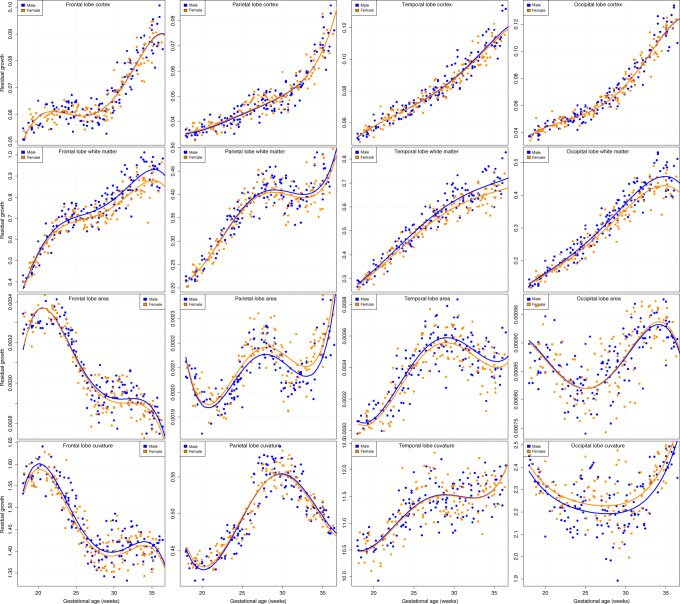
Residual lobe measure growth trajectories after accounting for global measures. Graphs of residual lobe measures of volume, surface area, and average absolute mean curvature after covarying for intracranial volume, total brain area, and average brain curvature, respectively. Model fits are shown for male and female data after transformation back to the measurement domain for visualization. The earlier expansion of the frontal lobe is followed by increases in parietal, temporal, and then occipital lobes over the 18 weeks of growth, each contributing to the global measure and resulting in a complex residual growth trajectory. The cortical tissue volume (top row) is seen to follow almost identical trajectories in male and female fetuses in all lobes, occupying the same proportion of ICV at all ages, while residual growth of white matter volume, surface area, and curvature diverge in different lobes.

Relative sizes of the regional anatomical measures in relation to global growth in the developing brain may indicate more fundamental differences in anatomy beyond overall head size effects. To assess for the presence of these differences, we modeled global and regional measures of volume, area, and curvature covaried by total ICV, total brain area (AREA), and average brain curvature (CURV), respectively. The data of the residual values and fitted models for the lobe measures are firstly summarized by the graphs in Fig. 3, which illustrate the more complex form (analysis details are given in the “Methods” section) of the relative growth compared to that of absolute growth. This reflects the different timing of the accelerated lobe growth over the period studied, combined with their contribution to the global measures that they are related to. As each lobe contributes a different portion of the whole brain measures, the relative trajectories show differently sized peaks at different times.

The first key observation is that after covarying for ICV, the cortical volume in each lobe follows a very similar trajectory in males and females (Fig. 3, top row). The trajectory of the frontal lobe cortex follows a trajectory with an initial peak, indicating at this point the accelerated absolute cortical growth (note the larger scale for frontal cortical volume in the third row of Fig. 2) in the frontal lobe is contributing a larger fraction of the whole cortex. After this peak at ~20–21 weeks, the cortical volume in other lobes begin to catch up, and the fraction of the whole cortical volume that is in the frontal lobe does not increase (and even slightly decreases) until ~27 weeks, where the frontal lobe cortical fraction again begins to increase further. Unlike cortical volume, there are more consistent male−female differences in relative white matter volume trajectories (second row of Fig. 3), particularly in the frontal, temporal, and occipital lobes. These plots of relative size also reveal complex trajectories reflecting the timing of accelerated growth of white matter in each lobe and the proportion that each contributes to the whole brain.

Relative surface area measures also follow differing trajectories in male and female brains, most noticeably in the parietal and temporal lobes. Relative surface area measures in this case will reflect the gross proportional size of underlying white matter in the simple early gestation brain, while later as the cortex folds, this will also reflect the relative timing of cortical folding in the lobes as this contributes to cortical area. Frontal lobe cortical area exhibits a large fraction of the whole area early in the developmental period, which then falls away as other lobes accelerate. The parietal cortex shows a peak in relative area at ~27 weeks. Relative curvature measures appear to follow similar trajectories in male and female brains, except for those of the occipital lobe, although there is also more variance in the measures derived from this smaller lobe region.

Statistical analysis of these models, summarized in Table 2, confirmed no statistical differences in the residual relative cortex volume measures either globally or for any lobe. White matter volume, however, was highly significantly (after correction for multiple comparisons) different both globally and for three of the four lobes. In contrast, the parietal lobe white matter volume showed no male−female difference after accounting for ICV. Cortical surface areas within each lobe also followed statistically identical trajectories in males and females after accounting for whole brain area, except the parietal lobe cortical surface area, which occupied a greater fraction (1.36% more) of the whole brain surface in females, with however statistical significance not surviving correction for multiple comparisons. Residual curvature of the brain surface in each lobe (i.e., testing for a lobe surface being more or less curved given the CURV) also followed a very similar trajectory in males and females, except for the occipital lobe. This exhibited significantly (after correction for multiple comparisons) greater curvature in females compared to males. All of the sex-related residual differences in the relative measures remained independent of gestational age, except the whole brain and frontal lobe white matter differences, which showed evidence for a statistically significant (uncorrected for multiple comparisons) change over time.

**Table 2:** Differences in residual relative growth trajectories.

R: Region	R_*M*_ − R_*F*_	Confidence interval		*P* value	*P* value
Measure	@ 27GW	−2.5%	+2.5%	(M∣F)	(M∣F) × AGE
CORT	0.03%	−1.47%	1.62%	9.69e − 01	8.76e − 01
WM	3.40%	1.48%	5.29%	**3.50e** **−** **04**^†^	**2**.**56e** **− 02**
DGM	0.36%	−1.75%	2.19%	6.83e − 01	2.41e − 01
CEREB	−0.18%	−2.64%	2.24%	8.94e − 01	7.11e − 01
Frontal CORT	0.10%	−1.49%	1.79%	9.00e − 01	9.02e − 01
Parietal CORT	−0.57%	−2.55%	1.58%	5.69e − 01	7.55e − 01
Temporal CORT	0.50%	−1.14%	2.11%	5.54e − 01	6.18e − 01
Occipital CORT	0.05%	−2.15%	2.33%	9.66e − 01	7.26e − 01
Frontal WM	3.71%	1.86%	5.55%	**3.81e** − **04**^†^	**1**.**92e** **−** **02**
Parietal WM	1.17%	−0.71%	3.14%	2.53e − 01	3.27e − 01
Temporal WM	4.31%	2.16%	6.44%	**4.93e** − **05**^†^	1.44e − 01
Occipital WM	4.67%	1.76%	7.43%	**1.34e** − **03**^†^	8.10e − 02
Frontal AREA	0.44%	−0.24%	1.14%	2.02e − 01	1.22e − 01
Parietal AREA	−1.36%	−2.19%	−0.51%	**4.48e** − **03**	1.66e − 01
Temporal AREA	0.88%	−0.21%	1.74%	6.24e − 02	6.19e − 01
Occipital AREA	−0.11%	−1.49%	1.39%	8.77e − 01	3.98e − 01
Frontal CURV	0.67%	−0.00%	1.37%	**4.39e** − **02**	9.84e − 01
Parietal CURV	−0.06%	−0.86%	0.80%	8.82e − 01	1.86e − 01
Temporal CURV	0.14%	−0.58%	0.89%	7.16e − 01	8.73e − 01
Occipital CURV	−1.66%	−2.58%	−0.60%	**1.38e** − **03**^†^	5.98e − 01

The estimated percentage increase from the female to male fetal brain (and the upper and lower 95% confidence bounds on this difference) of the residual increase of regional anatomical measures estimated after accounting for global differences in each measure (ICV for volume, whole brain AREA for lobe areas, average whole brain CURV for lobe CURV: see also Fig. 3).

*CORT* cortical tissue volume, *WM* combined tissue volume, *DGM* combined deep gray matter volume, *CEREB* cerebellum volume, *CURV* mean surface curvature averaged over the specified portion of the brain surface, *AREA* surface area of total brain or lobe region.

In addition, we include the statistical significance (uncorrected *P* value) of the male−female difference in the growth models and the significance of the change (interaction) of this with age over the study period. Bold indicates *P* < 0.05 for the male−female trajectory difference and dagger † indicates this significance survives correction for multiple comparisons across the 20 two-sided hypothesis tests in this table.

### Symmetry of growth trajectories

To examine differences in the way the left and right sides of the brain develop differently in males and females, we carried out a regression-based asymmetry test of the sex effect as summarized in Table 3. The only sex-related asymmetry difference that survived multiple comparison correction was in the frontal white matter, which exhibited a 0.98% greater volume asymmetry in males compared to females. A number of less statistically significant differences were seen in the global white mater volume, surface area and CURV, and in the surface curvature of the temporal and occipital lobes. Interestingly, there was a statistically significant (uncorrected) interaction in asymmetry in measures of both cortical volume and surface curvature in the occipital lobe, perhaps indicating evidence for the emergence of sex-related asymmetry with age.

**Table 3:** Differences in trajectories of brain asymmetry.

R: Region	*A*_M_ − *A*_F_	Confidence interval		*P* value	*P* value
Measure	@ 27GW	−2.5%	+2.5%	(M∣F)	(M∣F) × AGE
CORT	−0.13%	−1.00%	0.71%	7.57e − 01	6.69e − 01
WM	0.51%	0.06%	0.93%	**2.74e** − **02**	6.04e − 01
AREA	0.08%	0.01%	0.15%	**3.20e** − **02**	7.74e − 01
CURV	−0.43%	−0.76%	−0.03%	**1.73e** − **02**	5.17e − 01
Frontal CORT	−0.24%	−1.29%	0.74%	6.07e − 01	1.93e − 01
Parietal CORT	0.37%	−0.88%	1.65%	5.75e − 01	5.23e − 01
Temporal CORT	−0.46%	−1.64%	0.66%	4.30e − 01	3.48e − 01
Occipital CORT	0.29%	−1.14%	1.59%	6.90e − 01	**1**.**87e** **−** **02**
Frontal WM	0.97%	0.44%	1.57%	**4.62e** − **04**^†^	7.69e − 01
Parietal WM	−0.33%	−1.04%	0.34%	3.86e − 01	8.87e − 01
Temporal WM	0.50%	−0.48%	1.33%	2.91e − 01	9.63e − 01
Occipital WM	1.32%	−0.03%	2.75%	6.08e − 02	3.22e − 01
Frontal AREA	0.41%	−0.01%	0.85%	5.90e − 02	3.36e − 01
Parietal AREA	0.20%	−0.49%	0.87%	5.52e − 01	3.54e − 01
Temporal AREA	0.07%	−0.65%	0.74%	8.54e − 01	6.50e − 01
Occipital AREA	0.60%	−0.35%	1.52%	2.11e − 01	1.07e − 01
Frontal CURV	−0.12%	−0.71%	0.54%	6.78e − 01	8.26e − 01
Parietal CURV	−0.22%	−1.18%	0.78%	6.78e − 01	3.99e − 01
Temporal CURV	−0.69%	−1.35%	0.00%	**3.97e** − **02**	3.47e − 01
Occipital CURV	−1.29%	−2.23%	−0.44%	**4.28e** − **03**	5.74e − 02

The estimated sex-related difference A_M_−A_F_ in the asymmetry A = R_RIGHT_–R_LEFT_ from left to right, and the upper and lower 95% confidence bounds on this difference estimate.

*CORT* cortical tissue volume, *WM* combined white matter/developing white matter tissue volume, *DGM* combined deep gray matter volume, *CURV* mean surface curvature averaged over the specified portion of the brain surface, *AREA* surface area of total brain or lobe region.

The right two columns are the statistical significance (uncorrected *P* value) of this male − female difference and of its change with gestational age. Bold indicates *P* < 0.05 for the male − female trajectory difference and dagger † indicates that this significance survives correction for multiple comparisons across the 20 two-sided hypothesis tests in this table.

### Fine-scale voxelwise growth trajectories

**Fig. 4:**
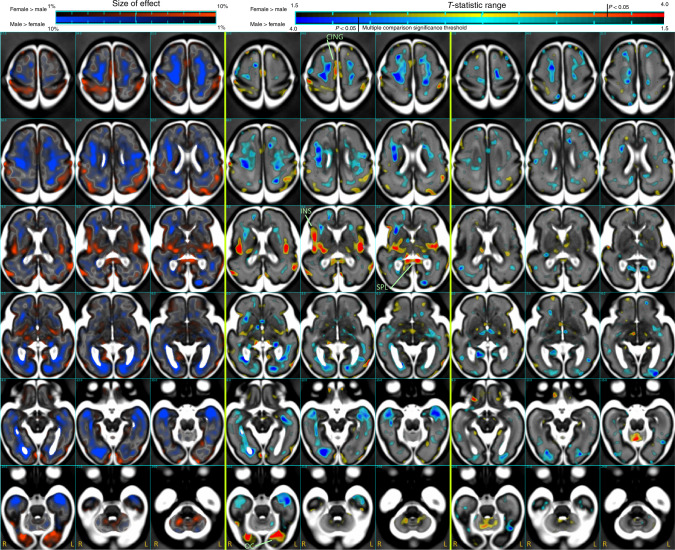
Differences in fine-scale volume growth trajectories after accounting for ICV. Replicating the analysis of Fig. 3 and Table 2 at a voxel level using deformation tensor morphometry: Axial slices (right in image is subject left) showing quantitative size (left column) and *T*-statistic maps (middle column) of the sex differences and any age interactions (right column), overlaid on the average MRI estimate at the average age (27.8 GWs). Blue-cyan indicates points where males have relatively larger volume over the 18 GWs after accounting for global ICV, and red-yellow indicates locations where females have relatively greater volume than males. For the *T*-statistic maps the darkest red and blue indicate those points surviving correction for multiple comparisons with two-sided significance (*P* < 0.05) marked on the upper right scale. Specific regions highlighted: CING: cingulate sulcus, OC: occipital cortex, SPL: splenium, INS: insular cortex.

To further examine differences in the growth of tissues over time, we employed tensor-based morphometry^30^ to map differences in fine-scale brain volume expansion patterns over all 18 weeks of growth. Here the growth trajectory of each voxel in a common anatomical space was estimated and tested for sex differences after global differences in ICV had been removed. This analysis, summarized in Fig. 4, revealed that regions where male brains were relatively larger than females are diffusely distributed throughout WM (blue) in frontal, temporal, and occipital regions, which refines the findings of the regional lobe analysis. However, some highly focal regions were found to be relatively larger in female fetuses compared to males. The most significant of these after correction for multiple comparisons were (in Fig. 4) in the splenium of the corpus callosum (SPL), the insular cortex (INS) (bilaterally, but with a larger anterior extent on the right), regions of the superior middle cingulate sulcus (CING) bilaterally extending to the superior frontal gyrus, and in locations around the parietal lobe cortex, including the postcentral sulcus, parieto-occipital sulcus, and intraparietal sulcus, and bilaterally in regions of the occipital cortex (OC). These regions were not quantitatively sufficiently large enough to impact the lobe region analysis, in which we found no overall differences in cortical volume after accounting for ICV.

Also, in contrast to the regional findings, we found small regions where there was a change over time in the volume differences between male and female (right column of Fig. 4). These were in frontal and occipital white matter where residual tissue volume difference toward males was increasing over time, and in regions of the parietal lobe cortex and white matter, where the residual volume differences were increasing toward females over time.

## Discussion

Global, regional, and fine-scale brain growth trajectories have been estimated using repeated measures in a large cohort of healthy human pregnancies with accurate, fine-scale morphometric tools. Unlike earlier smaller-scale studies often based on clinically scanned fetuses and without multiple repeated measures, we have been able to identify statistically significant evidence for the emergence and precise maintenance of differences in brain development in males and females long before birth. These findings confirm the very early prenatal presence of differences previously reported in the adult or pediatric brain. They also identify how sex differences and similarities change or are preserved during dynamic periods of neurogenesis and cortical folding.

The cortical gray matter volume differences of  +5.09% we found in fetuses is approaching that reported at  +8.2% in neonates^19^, and later in adults at  +7.7% in the recent large UK Biobank study^7^. Both of these other estimates fall within the 95% confidence intervals of our estimate. In the 4 weeks of in utero development remaining after our study period, and the 4 or more weeks of postnatal growth up to the study period of Dean et al.^19^, we can hypothesize from the literature and the graphs in Fig. 2 that continued rapid cortical tissue volume increases may be a prominent feature of development, as further surface folding occurs, and that this may account for increasing sex-related differences in global cortical volumes.

The global white matter differences we found in fetuses of  +7.37% closely match those reported in neonates of  +8.4%^19^, but are still appreciably lower than the  +13% reported in the large study of adults^7^. This later adult estimate also falls beyond the upper 95% confidence interval of our estimate. Later development, perhaps influenced by known further increases in hormones, such as testosterone during childhood^31^, may further drive changes in white matter proportions in a similar manner to white matter microstructure^32^, from the comparable fetal and neonatal levels, up to those seen in the adult brain.

Our estimate of  +6.24% greater total brain tissue volume in males compared to females is approaching the estimate of  +8.3% reported in neonates^19^, and is a significant step toward that reported in adults of  +10.5%^7^. The adult estimate again falls beyond the upper 95% confidence interval of our estimate . The magnitude of the differences observed in this study suggests that the residual differences in total brain volume between fetal and neonatal brains may be primarily accounted for by changes in the cortical differences discussed above.

Although we found differences in the mean absolute volumes of the cerebellum of +3.40% in our cohort, this difference did not survive correction for multiple comparisons and the confidence interval for this estimate remained wide, indicating that there may be unexplained variance that has not been accounted for. In addition, after accounting for ICV, there was no residual difference in the cerebellum volume (CEREB). This lack of strong statistical difference in the cerebellum agrees with previous neonatal findings at 1 month of age^18^, but is in contrast to the known differences in the childhood and adult cerebellums. For example, a longitudinal study of childhood development has found significant differences of as much as +10% starting at 5 years of age^33^, while a meta-analysis of adult studies^34^ reported absolute CEREB differences of ~+8.6% from female to male. Both of these estimates fall well beyond the upper confidence bound of our estimate. Growth of the cerebellum at the end of gestation^26,35^ and into the first year of life continues at a very high rate^36^, and therefore it may be the case that sex-related differences seen at 5 years and beyond emerge postnatally, much later than they do in the cerebrum volumes, and are perhaps modulated by later hormonal fluctuations.

In other regions, for example, deep gray matter, direct comparison to adults and even neonates is more difficult because of the challenge of extracting equivalent regions in fetuses across a range of gestational ages. This is due to lower or changing tissue contrast of some boundaries for many weeks of fetal development, and also partly because of the transient presence of neighboring developmental zones, such as the germinal matrix. We detected a difference in a combined region of deep gray matter of +3.50% in absolute volume, which did not survive statistical correction for multiple comparisons. In neonates individual measures that form part of our combined region have been reported, where Dean et al.^19^ found differences of ~+6.9% to +7.0% in the thalamus, +6.8% to +7.7% in the pallidum, +7.6% to +8.5% in the putamen, and conversely −8.2% to −8.0% in the caudate. All of these fall beyond the upper 95% confidence bound of our collective DGM estimate. The question of whether these are inconsistent with our fetal measures, however, may require a fetal study specifically limited to late gestation neuroanatomy that focuses on extracting adult corresponding subregions of the deep gray matter, rather than studying the longer growth trajectories of age-consistent anatomical MRI boundaries we have measured here.

Overall, these global comparisons indicate that a significant fraction of the tissue volume sex differences seen in the cerebrum in adults are established, but do not yet attain their adult magnitude, throughout the later half of pregnancy, while sex differences in other regions, such as the cerebellum, may emerge more strongly during postnatal growth.

Beyond differences in raw tissue volumes, there is clear evidence in our data for the controlled sculpting of tissues to create different regional anatomical proportions during the 18 weeks of growth that we studied. After accounting for ICV, there was no difference in global or lobe cortical volume or brain surface area. However, WM contributes to a significantly greater fraction of cranial volume in male fetuses. This appears to arise from greater relative volume in the frontal, temporal, and occipital lobes with, in striking contrast to these lobes, no statistical difference in the relative parietal WM volume. Conversely, the relative proportion of the brain surface area occupied by the parietal lobe is greater in female fetuses than males, which is not the case for the other lobes.

In neonates, a similar greater relative parietal lobe volume in females has been reported in a recent study^21^, after correction for total gray matter volume. In adults, it has been reported that there is no sex difference in the parietal lobe in either cortical gray matter or white matter volumes^37^, after correcting for global volumes. For surface curvature measures, the more recent larger neonatal studies have either not found or examined regional surface curvature differences, whereas in fetuses we found strong statistical evidence for greater occipital lobe curvature in females and (less significantly) greater temporal lobe curvature.

Early fetal brain asymmetry in cortical folding has been studied in normative clinical cohorts using manual measures in 2D slices^38^ and later using early motion corrected methods for fetal MRI^39^, and have identified statistically significant local differences in cortical folding in the left and right hemispheres before birth. Asymmetries were also detected in fetuses in local volume estimates in a tensor-based morphometry study over a smaller cohort and age range^30^. However, none of these studies reported statistically significant differences in asymmetry between males and females. Building on this work, our newer imaging measurements detected strong statistical evidence for sex-related differences in frontal white matter volume asymmetry, with greater asymmetry occurring in males, which remained proportionately unchanged with gestational age. This finding appears to agree, at least in general, with the reported presence of greater adult asymmetry occurring in males^40^. We also found some less statistically significant differences in asymmetry. Of these, the closest to surviving correction for multiple comparisons was the difference in the occipital lobe curvature. In addition, temporal lobe curvature asymmetry appeared significantly different, but only just below an uncorrected *P* < 0.05 level. Measures of asymmetry of global white matter volume, whole brain surface area, and average whole brain curvature were also significant, but again not approaching a level of surviving correction for multiple comparisons. Finally, there were age interactions in the sex-related asymmetry differences in occipital cortical volume. Overall, these observed effects were perhaps complimentary to the occipital curvature differences, reflecting greater male rightward cortical volume asymmetry and conversely lesser male rightward surface curvature asymmetry.

Tensor-based morphometry was able to detect fine-scale differences in growth trajectories that could not be detected using larger-scale region-based hypothesis testing. In particular, we observed consistently larger proportions of tissue volume in regions of the INS bilaterally in females, but with a larger anterior extent on the right. This appears to reflect findings in a recent study of adults who reported larger relative gray matter bilaterally in the posterior INS in a large (2838 sample) voxel-based morphometry study of adults^41^. The right INS was also reported to be larger in adult females after accounting for brain volume in the large 5216 subjects in UK Biobank study^7^ and in a voxel-based morphometry study^34^. In a recent study of fetal testosterone exposure, smaller gray matter in the anterior insula was associated with higher levels of fetal testosterone^42^. Interestingly, however, our finding disagrees with the results of a study of neonates^19^, which reported greater gray matter volume in males in the INS, hippocampal, and amygdalae after accounting for global volume.

Second, the volume growth trajectory maps also revealed evidence for a relatively larger size of the SPL in females in relation to male fetuses over the 18 weeks of growth studied. Many studies have explored differences in the corpus callosum anatomy in the male and female adult brain, and generally there is support for a greater relative overall size of the corpus callosum in adult women^43^. One of a limited number of developmental studies examining the corpus callosum reported regionally greater corpus callosum size in females, which remained after accounting for brain size, in neonates^19^. For the gestational period we studied, it is interesting to note that the larger relative splenium white matter in females may be linked to the more similar relative size of the neighboring parietal lobe white matter in males and females. In contrast, other lobes, associated with more anterior corpus callosum connections, show greater relative volume increases from females to males.

Third, we also detected evidence for greater relative cortical tissue volume in females in a region of the cortex in the CING extending into the superior frontal gyrus. This appears to agree with neonatal findings^19^ of larger tissue volumes in female neonates in the middle and anterior cingulate gyrus. However, in our fetal age range, we did not find evidence for the sex difference that was reported for neonates in the posterior cingulate gyrus^19^.

There are a number of anatomical measures that follow identical growth in male and female fetuses. The raw measures of CEREB are statistically indistinguishable, even without accounting for ICV differences. The development of cortical anatomy at a global and lobe scale after accounting for global measures also appears remarkably similar (as seen in Fig. 3). However, given the similarity of these measures, the large-scale differences in white matter proportions between male and female are, in contrast, also remarkable, as these represent a significant fraction of the differences reported in adults. Second, it is interesting that many of the differences in tissue proportions appear to be carefully maintained on a lobe scale as large changes in brain volume and shape occur, and the developmental machinery and processes of axonal outgrowth, glial proliferation, dendritic and synaptic development, and neuronal connectivity and circuit formation^44^ transform the brain into mature white matter and cortex. This may indicate that overall proportions may have been predetermined in earlier phases of hormone release and neurogenesis. Third, beyond head size effects, the parietal lobe white matter volume appears to follow a much more similar path of development in males and females than the other lobes (Table 2 and Fig. 3), which, in general, appear to grow relatively larger in male fetuses than in females. Conversely, parietal lobe area appears to occupy a proportionately larger area of the cortex in females than in males, although global area measures may be smaller. Finally, another interesting observation is the evidence for the emergence of some early differences in asymmetry between male and female fetal brains in the form of frontal white matter growth and some less significant differences in occipital lobe growth.

The significant differential effect of sex on white matter development we observe before birth could potentially be linked to reported differences in white matter connectivity patterns reported in adults^45^, where findings suggest that male brains may be more optimized for intrahemispheric communications, while female brains tend to be more optimized for interhemispheric communication. White matter tissue properties in adults have been shown to be related to exposure to sex hormones^32^, and in young adults, hormones have been shown possibly to be a factor in sex differences seen in regional white matter structure^46^.

Before birth it is believed that as part of normal sexual differentiation^47^, brain development may begin to differ at the scale of cellular organization in the male and female, due at least in part to androgen production^11^. GWs 8–24 have been postulated to be a critical period for the influence of testosterone^48,49^ on fetal development. Fetal testosterone levels have been hypothesized to peak between 14 and 18 weeks^13,14^. Studies of amniotic fluid measures of testosterone have indicated that the levels may then remain consistent over the remaining weeks of pregnancy^50^. This stable level may relate to our findings of consistent differential proportionate growth in males and females in many brain regions after 18 GWs.

In adults, the proportion of parietal lobe tissues has been found to be greater in females than males^51^, which our prenatal findings appear to agree with. Such differences found in adults have been linked to differing performance on the mental rotation test^37^. Postnatal human studies have found that in male–female twin pairs, female twins exhibited statistically better performance on this task^52,53^, which raises the possibility of twin–twin testosterone exposure modulating brain anatomy and later function. Our findings on prenatal parietal lobe development in males and females may fit in with the anatomical aspect of this hypothesis of testosterone exposure.

Our results examining differences in brain asymmetry indicate evidence that there are weaker statistical differences in asymmetry in total white matter, surface area, and curvature. There is stronger statistical support for regional differences in frontal white matter volume asymmetry and also occipital lobe surface curvature. There is also lesser statistical support for differences in temporal lobe curvature. Over the period of development, that we studied, there is also some statistical evidence of changes in the asymmetry differences in the OC tissue volume and surface curvature. It is interesting to note that our findings of frontal lobe and occipital lobe asymmetry differences may relate to the development of the known frontal–occipital torque in adult brain asymmetry^54^.

In conclusion, using the latest developments in neuroimaging and image analysis, these findings provide, to the best of our knowledge, the first clear statistical evidence in healthy human pregnancies of the dynamic emergence of early anatomical differences in male and female brain development and how they change over long periods of prenatal growth. Collectively, these findings indicate that it is not simply postnatal or childhood growth, or adolescent pruning of cells, that contribute to larger scale, and some finer scale, differences reported in adult and pediatric neuroanatomy. Sex-specific characteristics in the fetal brain go beyond simple global scaling effects to include the differential sculpting of brain regions at the scale of lobes and tissue types. These differences are already present half way through pregnancy and many are preserved during rapid cortical growth over the final half of gestation.

A practical message from this work is the importance of accounting for potential normal male − female differences in new anatomical studies of early human brain growth, either in utero or after premature birth. Further, from our complex growth trajectory estimates, it may also be advisable to maintain a sex–age balance in studies covering more than a narrow age range to account for the rapid changes occurring before normal term age. Finally, studies covering many weeks of growth, and that aim to examine the relative development in different regions of the brain, might consider the complex form of relative growth trajectories and the use of models that can account for changes in the variance in different developmental measures over time. Such study guidance is also applicable to imaging of early functional activity, which may rely on unbiased anatomical localization of functional signals, and thus may be susceptible to possible confounds arising when anatomical differences potentially impact functional data analysis.

It is important to note that, similar to studies of adults, our findings show differences in statistical distributions of neuroanatomical characteristics, and not binary definitions of neuroanatomy and, as with features in adult male and female brains, there is an overlap in the range of characteristics associated with potential male or female assignments. The studies that are now possible with modern imaging and image analysis techniques open up many new directions for research into this very early normal variation in brain anatomy, as well as factors such as maternal, environmental, hormonal, or genetic variables potentially affecting it. How these prenatal features may be related to postnatal growth and how they may be modified over the course of pregnancy are also promising directions for new studies using increasingly sensitive fetal neuroimaging techniques.

## Methods

### Cohort and recruitment

All research was carried out under a University of Washington IRB-approved protocol (#00001931). Healthy pregnant volunteers with a singleton pregnancy were recruited by web and paper flyer’s and email lists from clinics in the Puget Sound area and consented by trained recruitment staff at the University of Washington Institute for translational health sciences. Written informed consent was obtained from each pregnant volunteer before each entered the study. Participants were excluded if they had any contraindications for MRI scanning (including but not limited to: non-removable metal, medical implant, that is, heart valve, insulin pump, cochlear implant, electrodes or wires, stents, bone joint screws, pins, rods, an artificial limb, claustrophobia, excessive tattoos on stomach, back or abdominal area, breathing problems or motion disorder, or a history of being shot or injured by metal objects or impacted by shrapnel). Participants were excluded if they were too large to fit in the MRI system and remain comfortably for 45 min. Subjects were also excluded if they had congenital infection, known chromosomal abnormality (i.e., abnormal amniocentesis or chorionic villus sampling), or abnormal maternal triple screen (unless followed by normal amniocentesis). Subjects were also excluded during the study if they had later clinically identified fetal abnormalities or pregnancy complications.

A total of 165 subjects were recruited and scheduled for a total of 308 fetal MRI scans. Of these scan attempts, 162 subjects produced 268 morphometric measurements of acceptable quality for use in the study. To confirm normal development in each volunteer, all scans were visually inspected by an experienced fetal radiologist (Dr. Dighe) and their clinical follow-up was monitored to confirm a normal course for the pregnancy. One volunteer was excluded from the study because of a small for gestational age diagnosis made from clinical ultrasound carried out as part of normal pregnancy protocols. The final imaging cohort used in this study is summarized in Table 4 showing the male–female and age balance of the data collected. Only two factors showed evidence for a statistical difference between male and female cohorts (household income and household size), but with only a small to medium effect size. Evaluation of the effects on the morphometric analysis confirmed they did not significantly impact the findings.

**Table 4:** Summary of the fetal imaging data and cohort demographics (covering a range from a minimum of 18.0 to a maximum of 36.8 gestational weeks) used in this study.

Measure	All data	Male scans	Female scans	*T* statistic *P* value	Effect size
Num. of scans	268	139	129	–	–
Num. of fetuses	162	81	81	–	–
Num. of fetuses with one scan	88	41	47	–	–
Num. of fetuses with two scans	42	22	20	–	–
Num. of fetuses with three scans	32	18	14	–	–
Gestational age at scan (weeks)	27.63 (±5.12)	27.53 (±5.12)	27.74 (±5.14)	0.7456	0.040
Maternal age (years)	33.24 (±4.21)	33.25 (±3.69)	33.22 (±4.75)	0.9572	0.007
Paternal age (years)	35.50 (±5.96)	35.64 (±5.48)	35.34 (±6.45)	0.7019	0.049
Maternal height (m)	5.44 (±0.26)	5.46 (±0.26)	5.42 (±0.27)	0.1712	0.169
Maternal weight before (kg)	65.99 (±13.34)	66.67 (±14.50)	65.27 (±12.00)	0.4056	0.104
Household income (USD)	175 K (±124 K)	152 K (±92K)	200 K (±148 K)	**0.0052**	0.393
Num. in household	2.58 (±0.92)	2.42 (±0.74)	2.76 (±1.07)	**0.0044**	0.365
Num. of previous pregnancies	1.19 (±1.52)	1.07 (±1.34)	1.30 (±1.70)	0.2250	0.151
Num. of previous miscarriages	0.50 (±0.82)	0.46 (±0.84)	0.55 (±0.80)	0.3778	0.111
Num. of previous abortions	0.13 (±0.49)	0.13 (±0.57)	0.13 (±0.40)	0.9512	0.008
Previous ectopic pregnancies	0.02 (±0.15)	0.00 (±0.00)	0.03 (±0.22)	0.1027	0.211
Num. of previous still births	0.02 (±0.16)	0.02 (±0.15)	0.02 (±0.18)	0.7498	0.040

The overall and separate average (and standard deviation) for male and female cohorts are included for the contributing scans in each group. These are based on the subset of cases where the parents chose to provide answers. The only measures that exhibited statistically a two-sided difference (*P* < 0.05, uncorrected significance in bold) in means between the male and female scans that could potentially alternatively explain male vs. female differences in brain anatomy were the household income and number in household. Both of these were statistically greater for the female fetuses, but with only a small to medium effect size (Cohen’s *D*). We therefore explored the incorporation of these measures as covariates in the statistical analysis, and found that this did not significantly impact the findings. However, as not all participants provided responses to these questions, this would reduce the number of imaging measures; therefore, in the full cohort analysis we do not include these covariates.

### Fetal MRI

A key aim in imaging was to address fetal head motions. Fast multi-slice T2W structural imaging was used to study brain anatomy across the cohort and gestational age range. Imaging was carried out on a 1.5 T Phillips Achieva/dStream system (software release 5.1+) with a 16-channel receiver body coil. Multi-slice, single-short, fast-spin echo T2W (TE = 250 ms, TR = 1600–1800 ms, SENSE factor = 2) MRI with half Fourier readout was used to collect slices minimizing within-slice motion artifact. In-plane acquisition resolution was 0.75 × 0.75 mm^2^ (no interpolation or k-space padding) and the through-plane slice thickness was 3 mm. Three orthogonal slice plane acquisitions were planned and acquired in approximately axial, sagittal, and coronal planes with respect to the fetal head to provide complimentary directions of in-plane resolution. The three slice plane stacks were also repeated four times each using a dynamic acquisition approach to ensure adequate signal level and adequate coverage of fetal anatomy in each orientation in the case of large fetal head movements. The number of planned slices was selected in each case to account for the differences in head sizes, and also to minimize the chance of the fetal head moving out of the imaged volume during the acquisition. Repeat scans on the same fetus had a separation of between 4 and 6 weeks depending on the mothers availability and the clinical scanner availability. When processed, the collected scans were carefully examined for image quality and image segmentation accuracy and surface representation quality, yielding a combined database of repeated measurements summarized in Table 4.

### Motion correction and 3D image reconstruction

To assist replication of results, our automated data analysis steps whereever possible make use of common code and scripts that are stored and version tracked as part of an archive in our biomedical image computing group (BICG) source code control library, maintained over the past 20+ years. For the purposes of scientific record, we include the names of the primary software components used in the automated analysis described below, along with the version archive entry numbers used.

All image data were first imported into the SLIMMER tool^55^ for visual inspection and semi-automated setup of initial volume alignment for each acquired slice stack. After exporting the combined acquisitions from the SLIMMER tool, automated between-slice motion correction and 3D reconstruction was applied using a script (runHASTESIMC_FBD.csh V10978) on linux, running pre-compiled executable code for different steps: between-slice motion was estimated by full 3D slice motion correction using the conjugate gradient slice intersection motion correction (SIMC) approach^56^ (slimmerSIMCcg V8375). Three approximately orthogonal slice planes were collected, and using SIMC enforces an unbiased rigid alignment of brain anatomy in all the slices, which does not rely on an intermediate 3D reconstruction of the data as used by slice to volume approaches. Collectively, these steps ensure that the overall shape and size of the brain in the final 3D reconstruction after slice alignment is not distorted by through-plane movements during imaging, and volume contraction or expansion does not occur. To confirm the geometric integrity of this approach, we have collected careful repeated test–retest imaging in different cases of fetal head motion, and evaluated that the shape and volume measures resulting from the processing remain consistent between extreme motion and non-motion cases.

Intensity distortion arising from through-plane motion spin history-related artifacts were addressed in SIMC using a combination of outlier rejection for more extreme cases, and slice level intensity correction for more subtle cases^57^, which has been shown to improve the delineation of subtle developmental tissue contrast. Using the between-slice motion estimates, a single 3D motion corrected volume was created on a 0.5 × 0.5 × 0.5 mm^3^ voxel grid (to ensure adequate oversampling of the data) using a iterative reconstruction approach^29^ from scattered slices using a robust deconvolution of the acquisition point spread function (iterativeRecon V9800). This combined approach of between-slice motion correction and reconstruction has been refined over the past 10 years since the publication of the basic methods. Although more recent publications by other groups have explored different approaches to some of the problems we address, such as bias correction^58^, and regularization of deconvolution^59^ during reconstruction, these alternative techniques have so far not been applied or evaluated on larger-scale normative morphometry studies with repeated imaging, where robustness to large motions and geometric accuracy are key factors that our carefully refined imaging and morphometric pipeline provide.

### Age-consistent tissue segmentation and lobe parcellation

To study brain growth over 18 GWs, we used an age-consistent tissue boundary model to define and extract equivalent tissue boundaries consistently over the entire developmental trajectory. This was based on a separate manual tracing of reference MRI scans using the rview software (rview 9.077) segmentation tool, to define the anatomical boundaries. To impose region correspondence over the entire developmental period, we combined earlier transient tissue zones of the subplate, intermediate zone, and germinal matrix into a single region of presumptive developing white matter where these were visible in the MRI contrast. Cortical plate and later gray matter was delineated equivalently on each 3D reconstruction as the cortex. A coarse deep gray matter region was also delineated across all age ranges, as the individual structures of deep gray matter (including thalamus, putamen, and caudate) are not consistently visible in T2W contrast at all gestational ages. These definitions were used to create a carefully constructed age-consistent multi-atlas from 84 of the scans covering 18 to 36 GWs, at a voxel size of 0.5 × 0.5 × 0.5 mm^3^.

This multi-atlas data was used as a basis for automated subject-specific, multi-atlas patch-based segmentation of each MRI in the database using automated scripts to carry out age-specific atlas selection (SSMA7_Setup_FBD.csh V11026), alignment of the atlas data to the subject MRI scan (SSMA_Segment.m V11024) and multi-atlas label selection (runMAPLR_TissueLobe.csh V11025). To segment each scan at a given gestational age, all manually marked MRI atlas datasets within  ±2 GWs of the scan age were linearly and then non-linearly deformed to the space of the subject MRI data using a log demons diffeomorphic registration^60^. To ensure no left–right symmetry bias, atlas MRI were also additionally left-right reflected and separately aligned with the subject MRI to provide separate label estimates for the example-based patch search. To also ensure no male–female bias, the atlas subjects marked were selected to have an even distribution of male and female subjects over the age range studied and both male and female atlas subjects were used to automatically segment each subject dataset. Atlas mapping included a progressive label refinement that incorporated steps of bias correction and intensity normalization to remove variations in tissue signal level in the 3D reconstructions. Automated patch labeling^61^ (MultiAtlasPatchLabel V11024) was applied using the warped atlas datasets. We evaluated the automated segmentation of the 84 atlas scans using their separate manual tracings and achieved average tissue label DICE coefficients of (0.967 for WM, 0.901 for CORT, 0.953 for DGM, 0.953 for CEREB, and 0.932 for VENT) covering 18-36 GWs. A separate test of this pipeline was carried out by comparing automated segmentations using the pipeline to label 17 non-atlas datasets which had separate full 3D manual tracing of tissues. The average DICE coefficients on these separate studies were 0.958 for WM, 0.885 for CORT, 0.944 for DGM, 0.913 for CEREB, and 0.927 for VENT. All of these DICE coefficients were comparable with state-of-the-art accuracies seen in high-quality structural imaging of adult human brain anatomy, but spanning a wide range of fetal gestational age and brain size. The 84 atlas scans were themselves included in the study results, but to avoid bias from manual tracing of individual atlas scans, each of these was automatically segmented for the study without using the manual tracing from its own data.

In a similar way to tissue labels, a set of lobe regions (left-right: frontal, parietal, occipital, and temporal lobes and cerebellum) were marked on all 84 atlas scans. These were defined using cortical marks and extended down to provide a partitioning of white matter regions. To ensure consistency of partitioning at earlier gestational weeks, lobe definitions were carefully propagated from older to younger atlas subjects and then further manually refined to retain lobe scale partition proportions at the earliest gestation atlas times. Following the same procedure for tissue labeling, to label each new MRI, patch-based multi-atlas labeling was used to form the best estimates of lobe labels on each subject MRI scan. To further refine lobe labeling, automated labeling was applied to all 268 scans, and then carefully checked to visually and statistically examine the possibility for bias or mis-labeling of the regions. Further manual refinement of the atlas lobe label definitions was then applied as needed to ensure age consistency in the definition of anatomical boundaries.

### Surface extraction and characterization

Each of the tissue segmentations were used to create a topologically correct inner cortical surface mesh using an automated script (Tissues2SurfMesh_FBD.csh V9910) calling initial voxel label topology correction^62^ and then topology correct mesh creation from the label volume^63^ using an executable (vol2ssm V8941). The 3D surface locations were smoothed to remove voxel sampling artifacts^64^ while preventing shrinkage. The lobe labels on each subject MRI volume were then transferred to the subject surface mesh. The surface area of the whole mesh and each of the regions assigned each lobe label was then estimated by summing the triangular mesh areas falling in each label. For each point on the surface, the mean curvature of the surface was estimated at each vertex location using quadric models of the surface fitted to a patch of its two-ring vertex neighbors^65^. The average regional mean surface curvature was estimated by averaging the mean curvature over all vertices’s on either the whole brain (CURV) or in each labeled lobe region forming the frontal, parietal, occipital, and temporal lobe average curvatures.

### Image measurement quality checks and data exclusion

From 308 total scans intended to be acquired, 10 scans were not started or completed due to a subject discomfort, unannounced clinical scanner software configuration changes due to system maintenance, or power failures in the clinical center. Six more were lost to low signal to noise or extreme motion where inadequate data were collected for motion correction and 3D reconstruction. A further 24 were lost to later tissue segmentation errors mostly due to low signal to noise or excessive within-slice motion artifact. These were identified by outlier analysis of the image summary measures and tissue intensity statistics, followed by visual image and segmentation checks. The remaining 268 were then used for the morphometric analysis.

### Models of absolute growth

**Fig. 5:**
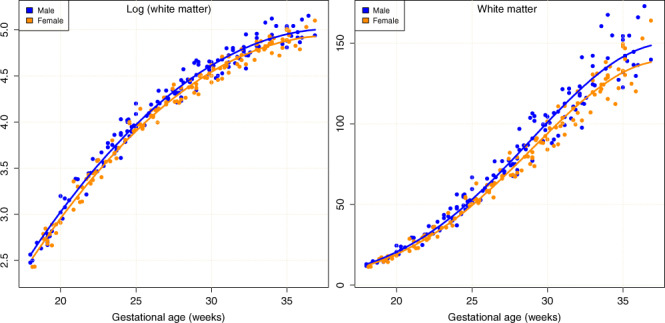
Plots of the male (blue) and female (orange) log-transformed datasets with separate mixed-effects estimates for each in (left) and then transformed back into the measurement domain (right). Note in the raw measures the increase in variance with age due to accumulated developmental differences, and the more age-independent variance in the log measurement domain. In the log domain there is a consistent fixed separation of the volume estimates. This corresponds in the right plot to a fixed fractional difference in volume estimates from female to male maintained throughout the gestational period (see Table 1 for specific numerical estimates).

Because biological growth is an inherently multiplicative process, variation in genetic and environmental conditions increases the variation among individuals in measures such as mass and volume through time^66^. This inherently induces heteroscedasticity in the measures against time. Visual inspection of plots of our measures confirmed the presence of heteroscedasticity in our data and we examined the data using standard approaches^67^ to confirm. To address heteroscedasticity, we use the standard approach of growth modeling and apply a logarithmic transformation to the measures^66^, which forces the multiplicative relationships to become additive, as illustrated for our total brain volume measures in Fig. 5.

Further inspection of the raw growth plots revealed a curve in the measures, indicating an acceleration of growth. There is a range of growth models developed and used for biological and population studies^66,68^ and also postnatal brain developmental models^69^. Most of these more complex models, however, are intended to address the eventual slowing of growth and convergence to a final stable size, for example, in postnatal and childhood growth. In fetal brain growth, even at the end of our study period, there is no convergence to a stable value in the measures. In addition, growth rates of the measures were either still increasing or only just beginning to slow, following the known peak brain growth rate pattern of fetal brain volume in humans^70^. Thus, more complex non-linear growth models were judged to be inappropriate for our data and time period. We took the approach for all models of selecting a simplest possible form, taking one step beyond a linear to a quadratic model. Such growth curves have previously been used both globally^25^ and locally^30^ in fetal MRI morphometry studies. We found that the second-degree polynomial fitted to the log-transformed data captured the change in growth rate well, so that for each measure *Q* we carried out a linear regression:

1 $$\mathrm{log}\,(Q)\approx a(M| F)+bW(M| F)+cW+d{W}^{2},$$

where *W* is the gestational week for scans that were scaled and centered using standard procedures in *R* for polynomial modeling. The estimated terms are: *a* the resulting sex effect, *b* the sex–age interaction, and *c* and *d* are the common growth terms with gestational week. This basic form was then recast for mixed-effects modeling to account for subject variance.

### Models of global relative/proportional growth

To examine differences in growth trajectories that are beyond overall head size effects in a way comparable with adult and pediatric studies of male − female differences, we aim to account for global growth (total ICV for volume measures, AREA for area measures, and the average whole brain curvature (CURV) to examine if the relative growth of parts of the brain differ in male and female. Rather than evaluate proportion ratios directly, which introduces a number of potential statistical challenges^71^, we use a covariate approach to examining the relative composition of the ICV as it is growing. The key factor in this task is not to introduce bias in the modeling that may arise from differences in global measures between male and female subjects. For region-based analysis, as we have here, analysis techniques to account for ICV in adult studies of sex differences were recently reviewed in Nordenskjöld et al.^72^, where a range of techniques were examined, including different normalization methods and incorporation of ICV as covariates. In our study, we have also the additional challenges of studying a period of rapid non-linear growth and also the task of consistently accounting for repeated measurements in each subject. We experimentally examined many of the proposed approaches on our data and selected the most general approach of covarying for global measures in the modeling, which can also be can be easily combined within the mixed-effects modeling framework for repeated measurements. As when studying absolute growth measures, we also need to select appropriate forms of relative growth with respect to age. After carefully inspecting data plots of residuals and ratios, we found that basic global proportions (e.g., total white matter to ICV) followed a curved form (as with the raw growth measures), which could be captured by a quadratic model of the log-transformed measures. Thus, for the relative analysis of all global measures *Q* ∈ {WM, CORT, DGM, CEREB, AREA, CURV}, we used a model of the form:

2 $$\mathrm{log}\,\left(Q\right)\approx a(M| F)+bW(M| F)+cW+d{W}^{2}+e\,{\mathrm{log}}\,(G),$$

where the normalization measure *G* is either the ICV for volume measures, AREA for area measures, or average whole brain curvature (CURV) for curvature measures. ICV was the sum of all the measured cerebral and cerebellum tissues and the VENT and SCSF measures. This basic form was then recast for mixed-effects modeling to account for subject variance.

To examine differences in developmental trajectories in relation to whole brain measures was more complex: inspection of the residuals confirmed that relative lobe measures or residuals relative to ICV followed a more varied trajectory, reflecting the contributions of the differently timed development of frontal, parietal, temporal, and occipital lobes to the whole brain measures used as a reference over the 18 GWs studied. In its simplest form, the different timings for these lobes impacting the global measure creates the possibility of up to three critical points in the relative trajectories, as the lobe developments accelerate at different times (see Fig. 3). We examined lower-degree polynomial models for relative growth and found that these often fit the simpler relative trajectories (e.g., the temporal and parietal cortical growth in Fig. 3). However, these failed to capture the critical points in many others, such as the frontal lobe cortex volume, parietal lobe white matter volume, and the lobar areas. Fitting different models to different parameter and lobe relative growth data would make comparison of results and significance more complex. We also examined non-parameteric, kernel-based approaches, but these can make mixed-effects modeling and significance testing more complex and difficult to interpret globally. We therefore selected the simplest polynomial model (a degree 4 polynomial) that adequately explained the most complex four-lobe form that occurred in the trajectories and report these for all the measures to allow the most direct comparison. The model used for all the lobes and measures *Q* such that:

3 $$\begin{array}{ccc}&&\mathrm{log}\,\left(Q\right)\approx a(M| F)+bW(M| F)+\\ &&cW+d{W}^{2}+e{W}^{3}+f{W}^{4}+g\,{\mathrm{log}}\,(G).\end{array}$$

As with global model fits, this was then recast in a form to incorporate subject and time point coding for mixed-effects modeling to account for repeated measurements in subjects when they occurred.

### Models and tests for asymmetric growth

There are a range of published measures used to evaluate brain asymmetry, particularly in adult studies that do not involve growth. These include indexes such as the laterality index^73^ and asymmetry index^74^. However, these can exhibit statistical limitations that become more important when analyzing them with more complex multivariate models. First, because these index measures form a ratio of proportionality between variables, they suffer from problems with the validity of the modeling of residuals^71^. Second, when there is the possibility for more complex relationships between multiple covariates present in the data, it has been pointed out that such ratio measures may inappropriately account for the variances and covariances^75^. Bullmore et al.^75^ suggested that it may be preferable to use a regression-based comparison of left and right measures^75^ to better account for these covariances. They proposed to first model the left and right sides in relation to covariates from the full sample of data and then analyze the residuals in the left and right estimates. In our case, we wish to account for significant non-linear growth and the effects of sex and its interaction with age. We therefore model the measure from the left side of the brain in terms of the growth as a function of age, the measure from right side of the brain (to account for asymmetry), and the global reference measure. We then add the two variables of interest, the sex effect and its interaction with age, to examine the remaining variance that was not explained by all other terms.

Thus, we extended the absolute growth model in Eq. (1) to model the left-side measures *Q*_LEFT_ with the right-side measures *Q*_RIGHT_ as a covariate such that:

4 $$\begin{array}{ccc}&&\mathrm{log}\,\left({Q}_{\mathrm{LEFT}}\right)\approx a(M|F)+bW(M|F)+\\ &&cW+d{W}^{2}+e\,{\mathrm{log}}\,({Q}_{\mathrm{RIGHT}})+f\,{\mathrm{log}\,(G)},\end{array}$$

so that the sex effect *a* captures any residual difference in the growth and *b* any changes of this residual with age and the factor *f* accounts for any residual covariance with global measure *G*. From the model fit, we can then also estimate the average difference in the asymmetry for male and female for a given age.

### Model fitting to repeated measures with significance testing

All summary statistical measures for each global and regional measure for each subject were analyzed using standard models written in the R statistical package^76^ version 3.6.0 (2019-04-26). To account for and make use of repeated measures in the regression, we used mixed-effects methods that have been employed in other long-term brain development studies^77^. Here we used the *lmerTest* package in R^78^ to estimate fixed and random effects across subjects and provide estimates of significance in an unbalanced study (taking into account the different numbers of time points and gestational ages of the scans in different fetuses). Subject ID and sex were encoded as factors in the mixed-effects regression model. Fitting was achieved using REML, and *p* values were derived using the Satterthwaite approach as suggested in Luke^79^. For each difference estimate, the upper and lower bounds for the 95% confidence interval were estimated using a bootstrap approach to account for fixed and random effects in the models in log measure domain and then these were transformed to the measurement domain for interpretation. Because of the need to employ higher-degree polynomial models to capture the relative growth of lobe measures over time, and the limited number of time points collected in each subject, there is a limit in the ability to model within-subject growth using higher-degree polynomials. However, because we chose a relatively small time interval (of ~4 weeks) between the repeated measures in a subject, within-subject growth can be modeled using a lower-degree polynomial covering the up to 3 measurements collected in each subject. This was carried out within the R package, which reduces the number of within-subject polynomial coefficients to match the measurements available.

### Deformation tensor morphometry

To examine local growth trajectories, we applied deformation tensor morphometry of the fetal brain MRI scans and tissue maps^30^. We applied unbiased symmetric groupwise log domain diffeomorphic demons alignment^60^ of all data to provide accurate alignment across large scales of growth. This was applied in an unbiased groupwise framework, whereby all scans were collectively non-linearly aligned to form a single unbiased average anatomical space during registration, which forms the geometric shape average equidistant to all the scans. This was implemented in C++ (GroupSeqReg V10794). From the set of spatial transformations we estimated, the Jacobian determinant ∣*J*(**x**)∣ of the mapping at each point **x** in this group average to each subject scan, to describe the relative anatomical size of each point in the common space in relation to its anatomical location in each individual scan. The resulting maps of local volume change were smoothed with a 4-mm full width at half maximum (FWHM) Gaussian kernel, log transformed, and then fit with a polynomial of degree 4, in a manner to match as closely as possible the regional lobe analysis above, such that:

5 $$\begin{array}{ccc}&&\mathrm{log}\,\left(| J({\bf{x}})| \right)\approx a(M| F)+bW(M| F)+\\ &&cW+d{W}^{2}+e{W}^{3}+f{W}^{4}+g\,{\mathrm{log}}\,(G),\end{array}$$

where the global covariate *G* in this case summarizes the global size changes from average common anatomy to each subject in the form of the spatially average Jacobian determinant $G=\overline{| J({\bf{x}})| }$. This is averaged over the ICV in the common anatomical space, leaving the factor *a* to be the sex effect of interest. *T*-statistic maps of the sex difference were then estimated and corrected *T*-statistic thresholds for each voxel location were calculated using non-parametric permutation correction methods^80^, using 20,000 permutations of the scan covariates to build cumulative distributions of the *T*-statistics. This voxel-based analysis was implemented run using a standard script (runScalarTBM_FBD.csh V10923) calling executables written in C++ (SimpleVoxSPM V10925) for multi-threaded statistical analysis.

### Reporting summary

Further information on research design is available in the Nature Research Reporting Summary linked to this article.

## Supplementary information

Reporting Summary

## Data Availability

All summary measures used for statistical analysis in Tables 1, 2, and 3 and Figs. 2 and 3 are stored in CSV format for analysis in the R package. These data can be made available upon reasonable request to the first author given the constraints imposed by institutional regulations at the University of Washington. Primary imaging data files, originally collected at the University of Washington Medical Center, may contain components governed by regulations on anonymity, or the intellectual property rules of the University of Washington, and will be shared in part on reasonable request to the first author, within the limits imposed by the institutional regulations and protocols. We are also happy to share expertise and collaborate with researchers aiming to collect their own data on different imaging systems.

## Code availability

The data analyses in the paper made use of both generic open source code (available for optional installation and maintenance as packages wthin linux) including R version 3.6.0 (2019-04-26) (https://www.r-project.org/) with the lmerTest package (https://cran.r-project.org/web/packages/lmerTest/index.html), C and C++ code written using the gnu g++ (Version 4.8.5) environment (https://gcc.gnu.org), and scripts written in csh and bash (https://www.gnu.org/software/bash/manual) shells and make (https://www.gnu.org/software/make), installed as part of standard linux environments. Custom code components written in gnu C and C++ in our Biomedical Image Computing Group (BICG) source code control library collectively implement algorithms described in previous work we referenced in the “Methods” section. These code components may be partially reliant on separate binary libraries or are specific to MRI file formats acquired on our scanner system (such as slice timing and ordering information not fully supported in DICOM image formats) and may therefore require significant changes to use with other image data. Custom code segments will be made available on reasonable request to the first author, which are permitted within the rules and protocols governing intellectual property at the University of Washington and the University of Washington Medical Center. We are also happy to share expertise and collaborate with researchers developing their own implementations of our processing pipelines using other computing environments and MRI data formats.
