# Supplementary material for: Motion corrected MRI differentiates male and female human brain growth trajectories from mid-gestation
Source: Nat Commun. 2020 Jun 16;11:3038. doi: 10.1038/s41467-020-16763-y (PMC7297991; doi:10.1038/s41467-020-16763-y)
Supplement: Supplementary file 1 — Reporting Summary [file 41467_2020_16763_MOESM1_ESM.pdf]

nature research

Corresponding author(s): Colin Studholme

Last updated by author(s): May 8, 2020

Reporting Summary

Nature Research wishes to improve the reproducibility of the work that we publish. This form provides structure for consistency and transparency in reporting. For further information on Nature Research policies, see Authors & Referees and the Editorial Policy Checklist .

Statistics

For all statistical analyses, confirm that the following items are present in the figure legend, table legend, main text, or Methods section.

n/a	Confirmed	
☐	☒	The exact sample size (*n*) for each experimental group/condition, given as a discrete number and unit of measurement
☐	☒	A statement on whether measurements were taken from distinct samples or whether the same sample was measured repeatedly
☐	☒	The statistical test(s) used AND whether they are one- or two-sided<br>*Only common tests should be described solely by name; describe more complex techniques in the Methods section.*
☐	☒	A description of all covariates tested
☐	☒	A description of any assumptions or corrections, such as tests of normality and adjustment for multiple comparisons
☐	☒	A full description of the statistical parameters including central tendency (e.g. means) or other basic estimates (e.g. regression coefficient) AND variation (e.g. standard deviation) or associated estimates of uncertainty (e.g. confidence intervals)
☐	☒	For null hypothesis testing, the test statistic (e.g. *F*, *t*, *r*) with confidence intervals, effect sizes, degrees of freedom and *P* value noted<br>*Give P values as exact values whenever suitable.*
☒	☐	For Bayesian analysis, information on the choice of priors and Markov chain Monte Carlo settings
☒	☐	For hierarchical and complex designs, identification of the appropriate level for tests and full reporting of outcomes
☐	☒	Estimates of effect sizes (e.g. Cohen's *d*, Pearson's *r*), indicating how they were calculated

Our web collection on statistics for biologists contains articles on many of the points above.

Software and code

Policy information about availability of computer code

Data collection	Data collection was carried out using a 1.5T Phillips Achieva/ dStream MRI system, with software release level of 5.1+ during the study period
Data analysis	The data analyses in the paper made use of both generic open source code using R version 3.6.0 (2019-04-26) (https://www.r-project.org/) with the lmerTest package (https://cran.r-project.org/web/packages/lmerTest/index.html), C and C++ code written using the gnu g++ (Version 4.8.5) environment (https://gcc.gnu.org) and scripts using csh and bash (https://www.gnu.org/software/bash/manual) shells and make (https://www.gnu.org/software/make/), installed as part of standard linux environments. Custom code components written in gnu C and C++ in our Biomedical Image Computing Group (BICG) source code control library collectively implement algorithms described in previous work we referenced in the Methods section. These code components may be partially reliant on separate binary libraries or are specific to MRI file formats acquired on our scanner system (such as slice timing and ordering information not fully supported in DICOM image formats) and may therefore require significant changes to use with other image data. Custom code segments will be made available on reasonable request to the first author, that are permitted within the rules and protocols governing intellectual property at the University of Washington and the University of Washington Medical Center. We are also happy to share expertise and collaborate with researchers developing their own implementations of our processing pipelines using other computing environments and MRI data formats.

For manuscripts utilizing custom algorithms or software that are central to the research but not yet described in published literature, software must be made available to editors/reviewers. We strongly encourage code deposition in a community repository (e.g. GitHub). See the Nature Research guidelines for submitting code & software for further information.

Data

Policy information about availability of data

All manuscripts must include a data availability statement. This statement should provide the following information, where applicable:

- Accession codes, unique identifiers, or web links for publicly available datasets
- A list of figures that have associated raw data
- A description of any restrictions on data availability

All summary measures used for statistical analysis in Tables 1, 2 and 3 and Figures 2 and 3 are stored in CSV format for analysis in the R package. This data can be made available upon reasonable request to the first author given the constraints imposed by institutional regulations at the University of Washington. Primary imaging data files, originally collected at the University of Washington Medical Center may contain components governed by regulations on anonymity, or the intellectual property rules of the University of Washington, and will be shared in part on reasonable request to the first author, within the limits imposed by the institutional regulations and protocols. We are also happy to share expertise and collaborate with researchers aiming to collect their own data on different imaging systems.

Field-specific reporting

Please select the one below that is the best fit for your research. If you are not sure, read the appropriate sections before making your selection.

[x] Life sciences [ ] Behavioural & social sciences [ ] Ecological, evolutionary & environmental sciences

For a reference copy of the document with all sections, see nature.com/documents/nr-reporting-summary-flat.pdf

Life sciences study design

All studies must disclose on these points even when the disclosure is negative.

Sample size	The sample size was not known prior to the study. The data analyzed was collected as part of normal cohorts in two separate NIH studies into methods for fetal brain imaging. This data was pooled for the purposes of the analysis in this paper. Statistical modeling accounting for multiple covariates in the population is used to ascertain the significance of the resulting contrasts that are reported.
Data exclusions	The approach to data exclusion is described in the methods section of the paper. Exclusion begins with cohort selection using per-established criteria, where we did not include cases of known or suspected clinical abnormality. The next stage of data exclusion is based on scan quality using measures of noise and image artifact as the repeated scans are being combined to form a 3D image. Finally, data is excluded after automated image segmentation based on metrics for image consistency in labeled tissues and outlier analysis of the measures which are then confirmed as tissue segmentation errors by visual inspection using per-established procedures.
Replication	We have used all the data we acquired for the modeling. We do not have the resources to carry out a replication study.
Randomization	No randomization of the cohort data was used. All collected data was used for the modeling and statistical analysis using mixed effects linear modeling using a maximum likelihood framework to account for multiple covariates.
Blinding	Measurement methods were automated and therefore users did not need to be blinded.

Reporting for specific materials, systems and methods

We require information from authors about some types of materials, experimental systems and methods used in many studies. Here, indicate whether each material, system or method listed is relevant to your study. If you are not sure if a list item applies to your research, read the appropriate section before selecting a response.

Materials & experimental systems

n/a	Involved in the study	
[x]	[ ]	Antibodies
[x]	[ ]	Eukaryotic cell lines
[x]	[ ]	Palaeontology
[x]	[ ]	Animals and other organisms
[ ]	[x]	Human research participants
[x]	[ ]	Clinical data

Methods

n/a	Involved in the study	
[x]	[ ]	ChIP-seq
[x]	[ ]	Flow cytometry
[ ]	[x]	MRI-based neuroimaging

Human research participants

Policy information about studies involving human research participants

Population characteristics	The subjects were volunteers from the Puget Sound area with some wider recruits from more remote Washington state locations. The study population therefore represents the socioeconomic mix of that area. We list the key characteristics of the pregnant cohort that was studied in Table 4. The results may potentially not be applicable to other populations.
Recruitment	Recruitment of pregnant volunteers was carried out as described in the methods of the paper from the Puget sound area clinics. All subjects were volunteers who therefore chose to enter the study out of there own interest in the development of their child. This, as with many studies of normal brain anatomy and function, may potentially may bias the cohort.
Ethics oversight	All protocols for imaging and data handling were approved by the University of Washington Institutional review board Committee on Human Subjects Research,. All pregnant subjects provided written consent before entering the study. Details are given in the methods section of the paper.

Note that full information on the approval of the study protocol must also be provided in the manuscript.

Magnetic resonance imaging

Experimental design

Design type	T2W multi slice structural imaging
Design specifications	Single shot fast spin echo half Fourier acquisitions (SSFSE) planned in three approximately orthogonal planes
Behavioral performance measures	These were not collected yet as not all fetal subjects have reached an age where testing can be applied.

Acquisition

Imaging type(s)	Structural
Field strength	1.5T
Sequence & imaging parameters	Multi slice HASTE imaging parameters were as described in the methods section of the paper.
Area of acquisition	fetal head

Diffusion MRI ☐ Used ☒ Not used

Preprocessing

Preprocessing software	Image preprocessing for motion correction is described in the methods section using published and referenced methods.
Normalization	All data were spatially normalized to a common average anatomical space using a non-linear unbiased groupwize alignment described in the methods section. (an implementation of the symmetric demons approach)
Normalization template	Unbiased Template Free Normalization was used for Tensor Based Morphometry
Noise and artifact removal	All 3D images were reconstructed using iterative methods described in the methods section.
Volume censoring	Large field of view 2D multi slice imaging was used.

Statistical modeling & inference

Model type and settings	As described in the methods section, Mixed effects models were used as implemented in the lmerTEST package in R. REML was used for fitting.
Effect(s) tested	All models tested the difference in growth trajectories fitted to measures from male and female brains.

Specify type of analysis: ☐ Whole brain ☐ ROI-based ☒ Both

Anatomical location(s)	Tissue classes globally and parcellated by the main lobes of the fetal brain as listed in the online methods section of the paper.
Statistic type for inference (See Eklund et al. 2016)	As described in the methods section, Non Parametric Permutation testing was used to correct T statistics maps of morphometric differences at a voxel level. No cluster assumptions were used.

Correction

Bonferroni correction levels are stated for each of the tables of B values and permutation testing was used for correction of spatial statistical maps.

Models & analysis

n/a	Involved in the study
☒	☐ Functional and/or effective connectivity
☒	☐ Graph analysis
☐	☒ Multivariate modeling or predictive analysis

Multivariate modeling and predictive analysis

Each multivariate linear model used is described in equation form in the online methods section of the paper.
